# Around the Hospitals

**Published:** 1893-08-19

**Authors:** 


### Around the Hospitals.

Notice to the Managers of Institutions.—Special space is now reserved for the insertion of notices of hospital meetings and festivals. The Editor requests that all notices may reach The Hospital Office, 428, Strand, at least one week before the date of each meeting.

**The Paddington Green Children's Hospital.**—We congratulate Mr. Pearce, the secretary of this institution, on the production of that rarity, an interesting report. We would not for a moment desire that all institutions should pass through the troubles and inconveniences which the Paddington Green Hospital has endured, and which have made it possible for something of a tale to be told as well as an official report to be given, but we could wish that all reports were produced in the lively and instructive manner which Mr. Pearce has used to clothe his facts. We learn that the Children's Hospital, homeless as far as Paddington Green is concerned, is doing well at Wealdstone, near Harrow. A house was taken there on the desertion of the old hospital, condemned as insanitary. This house it is hoped to retain as a convalescent home when the patients are once more located in the new hospital that is to be on the original site at Paddington Green. The sum necessary for these new buildings is £10,000 for the structure alone. Of this over £7,000 has already been subscribed. It is most desirable from the point of the larger amount of good, and economy, that the hospital should be commenced forthwith, and so it is to be hoped the committee will have little difficulty in raising the required sum to commence operations. Judging from the very large number of those who have made donations in kind to the hospital, an unusual amount of interest is taken in it. The plans of the proposed institution show that the hospital of the future will be a great improvement in every way on the hospital of the past.

**Sunderland Infirmary.**—A very satisfactory report comes from the Sunderland Infirmary, the past year having been one of exceptional activity. More patients have been treated than in any former year, and notwithstanding this increase of numbers, the rate of mortality has been materially lowered. We see that the total number of patients treated was 5,318, as against 4,712 last year, an increase of 606. The opening of the Heatherdene Convalescent Home in September last has proved a great blessing, many patients having been sent there during the year, and various members of the Infirmary nursing staff also have found a stay at the home very beneficial. There has also been a slight increase in income, and the Committee acknowledge with much pleasure and satisfaction the generous subscription standing in the name of the working men, amounting to the large sum of £4,759. Several structural alterations have been carried out, certain additions having been rendered necessary by the very considerable increase in the number of patients. A special item in the expenditure relates to fire extinguishing apparatus, a wise outlay on the part of the Committee. We are glad to see that the Committee express special thanks to the medical staff, the sisters, and nurses for their valuable services during the year, and recognition is also accorded to colliery owners for gifts of coal, and timber merchants and contractors for gifts of firewood. These gifts in kind are valuable additions to the resources of a hospital, especially where the selection made is a judicious one.

